# Drug‐Induced Phototoxicity in Vitiligo: The Role of Hydrochlorothiazide in Photosensitivity Dermatitis

**DOI:** 10.1155/crdm/4302190

**Published:** 2025-12-26

**Authors:** Andres D. Parga, Celina Dubin, Donald Rudikoff

**Affiliations:** ^1^ Department of Dermatology, BronxCare Health System, Bronx, New York, USA, bronxcare.org; ^2^ Department of Dermatology, Icahn School of Medicine at Mount Sinai, New York, New York, USA, mountsinai.org

**Keywords:** cutaneous photodamage, drug-induced photosensitivity, erythematous papules, melanocyte dysfunction, photosensitivity dermatitis, thiazide-induced phototoxicity, ultraviolet (UV) hypersensitivity, vitiligo

## Abstract

Photosensitivity dermatitis is a multifactorial dermatologic condition characterized by an exaggerated cutaneous response to ultraviolet (UV) radiation, often exacerbated by exogenous agents, particularly photosensitizing medications. The pathophysiology involves direct phototoxic or immune‐mediated photoallergic mechanisms, leading to inflammatory skin reactions upon UV exposure. In individuals with vitiligo, the absence of melanocytes in depigmented areas significantly diminishes photoprotective mechanisms, rendering these regions highly susceptible to UV‐induced damage. Consequently, vitiliginous skin is inherently more vulnerable to photo‐induced inflammation and cutaneous injury. We report a 54‐year‐old man with generalized vitiligo who developed erythematous papules confined to sun‐exposed depigmented skin. The clinical course suggested a medication‐induced phototoxic reaction, likely potentiated by the patient’s underlying lack of cutaneous melanin. This case highlights the need to recognize drug‐induced photosensitivity in vitiligo and understand how UV vulnerability and photosensitizing medications interact.

## 1. Introduction

Photosensitivity reactions encompass a diverse spectrum of dermatological disorders where the skin exhibits an exaggerated or abnormal response to UV radiation. These reactions can be classified into phototoxic and photoallergic responses, depending on their pathophysiology. Phototoxic reactions occur when a medication or chemical absorbs UV radiation and generates reactive oxygen species (ROS), leading to direct cellular damage. In contrast, photoallergic reactions are immune‐mediated, where UV light alters a drug’s structure, converting it into a hapten that triggers a delayed hypersensitivity response. Both types of photosensitivity can manifest as erythema, hyperpigmentation, pruritus, or blistering, often mimicking other inflammatory dermatoses [1]. These reactions are particularly problematic for individuals with vitiligo, a chronic autoimmune skin disorder characterized by the loss of functional melanocytes, resulting in depigmented macules and patches [2]. Melanin normally protects against UV‐induced oxidative stress; its absence in vitiligo increases susceptibility to photosensitivity [3]. Several medications, including antibiotics, NSAIDs, antifungals, diuretics, and chemotherapeutics, can exacerbate photosensitivity. These medications can amplify cutaneous damage by lowering the skin’s threshold for UV‐induced inflammation. In patients with vitiligo, who already face an increased risk of phototoxic injury, exposure to these drugs may result in atypical or severe presentations of photosensitivity dermatitis [2]. This case study outlines the symptoms, differential diagnosis, and treatment approach for photosensitivity dermatitis in a patient with vitiligo, further shedding light on how certain medications may contribute to these reactions. Given the growing prevalence of polypharmacy in dermatologic patients, it is critical to enhance awareness of drug‐induced photosensitivity, particularly in populations with heightened UV vulnerability. Further exploration of these mechanisms is crucial for advancing targeted measures and optimizing treatment approaches.

Here, we describe a case of a middle‐aged man with vitiligo who developed phototoxic dermatitis triggered by hydrochlorothiazide (HCTZ), underscoring the interplay between drug‐induced photosensitivity and melanocyte absence in depigmented skin.

## 2. Case Presentation

A 54‐year‐old man with a history of vitiligo presented to the dermatology clinic for a follow‐up evaluation after experiencing persistent skin changes that initially mimicked a fungal infection. The patient first noticed well‐demarcated hyperpigmented patches on his upper back and chest, which were suspected to be tinea versicolor. However, despite treatment with ketoconazole shampoo and clotrimazole cream, the condition did not improve. Given the lack of response to topical antifungal therapy, the patient was prescribed systemic antifungal agents, including fluconazole and griseofulvin, but continued to experience no improvement. This lack of therapeutic response raised suspicion of a noninfectious etiology. During follow‐up, the patient reported new symptoms, including the development of pruritic, erythematous papules localized to sun‐exposed areas, particularly the dorsal hands and face, occurring in regions of depigmented vitiligo skin (Figures 1(a) and 1(b)). The patient noted that the rash consistently appeared after sun exposure but did not resolve with the regular application of sunscreen. The distribution and persistence of lesions suggested a photosensitivity reaction rather than a recurrent fungal infection or primary inflammatory dermatosis. A thorough review of the patient’s medication history identified olmesartan‐HCTZ, a well‐documented photosensitizing agent, as a potential contributing factor (Table 1). HCTZ, a diuretic commonly used in combination antihypertensive medications, has been associated with drug‐induced photosensitivity due to its ability to absorb UV radiation and generate ROS. The patient was also on prednisone for an inflammatory condition and hydroxychloroquine (Plaquenil) for systemic lupus erythematosus (SLE) immunomodulation. Notably, Plaquenil has been implicated in exacerbating photosensitivity reactions in certain patients, although it is more commonly linked to photo‐distributed hyperpigmentation rather than acute photosensitivity dermatitis.

Figure 1(a) Erythematous papules on the dorsal aspect of the patient’s left hand, superimposed on depigmented skin patches. (b) Distribution of erythematous papules on dorsum bilateral hands.

**Figure:**
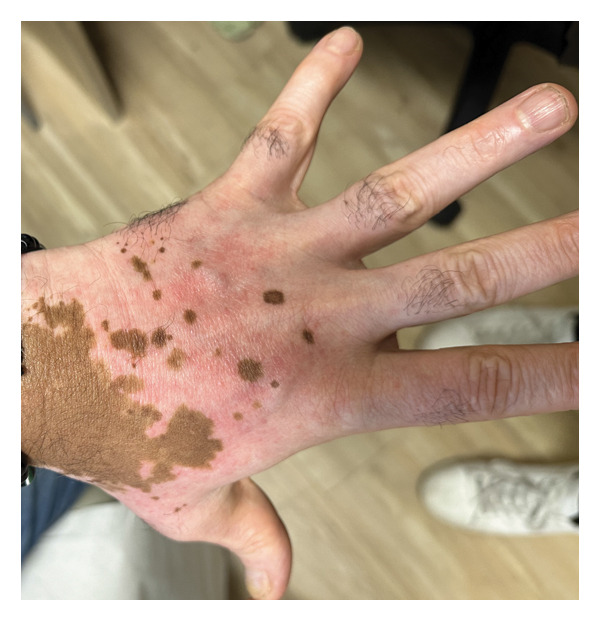
(a)

**Figure:**
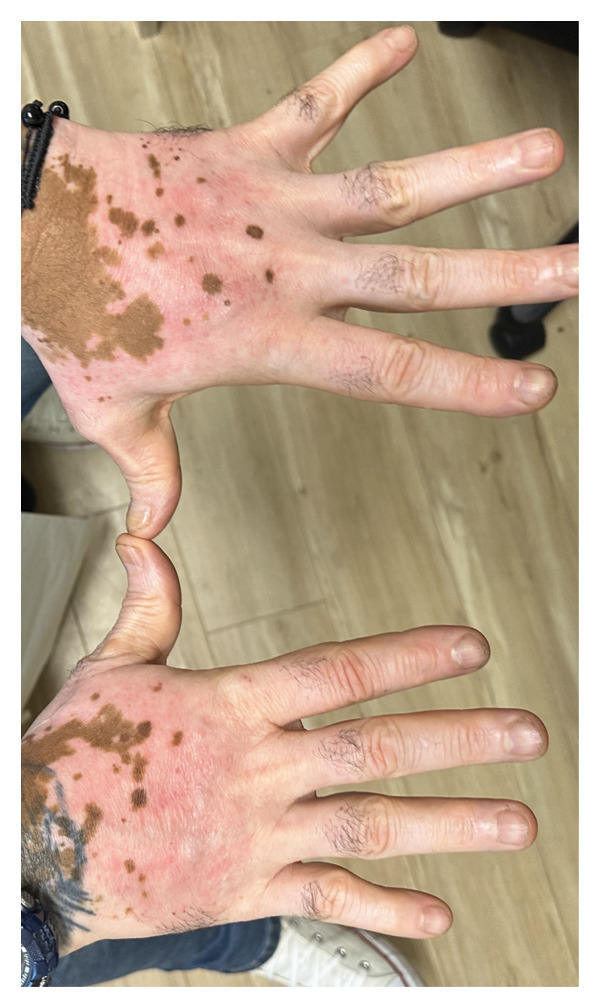
(b)

**Table 1:** Active medications and relevant history.

Medication	Dosage	Indication
Olmesartan‐hydrochlorothiazide	20 mg/12.5 mg daily	Hypertension
Prednisone	70 mg orally, taper	Inflammation control
Plaquenil	200 mg daily	SLE immunomodulation

Given the patient’s clinical history and the nature of his cutaneous findings, medication‐induced photosensitivity in the context of vitiligo was considered the most likely diagnosis. The presence of pruritic papules localized to areas of depigmented skin further supported the role of heightened UV susceptibility due to the absence of melanocytes. The patient was advised to discontinue olmesartan‐HCTZ and was counseled on strict photoprotection measures, including physical sunblock, UV‐protective clothing, and behavioral modifications to minimize sun exposure.

## 3. Discussion

This case highlights the clinical challenge of managing photosensitivity dermatitis in vitiligo, particularly when triggered by photosensitizing agents. Given the patient’s medical history, the suspected offending agent was olmesartan‐HCTZ, a combination antihypertensive medication known to induce photosensitivity reactions.

Photosensitivity reactions can be broadly classified as phototoxic or photoallergic. Phototoxic reactions, which occur more frequently, involve a nonimmunologic, dose‐dependent response in which UV light interacts with a drug or its metabolite to generate ROS, leading to direct cellular injury. In contrast, photoallergic reactions represent a delayed‐type hypersensitivity response, where UV radiation converts the drug into an antigenic form, stimulating a T‐cell‐mediated immune response [4]. The rapid onset of symptoms following sun exposure, coupled with the absence of systemic involvement or chronic eczematous changes, strongly suggests a phototoxic mechanism in this case rather than a photoallergic reaction. The patient’s presentation illustrates how the absence of epidermal melanin in vitiligo can intensify ultraviolet‐mediated oxidative injury, thereby amplifying the phototoxic potential of photosensitizing drugs such as HCTZ [1, 4–8].

## 4. Role of Melanin in Photoprotection and Vitiligo’s Increased Susceptibility to Photodamage

Melanin plays a crucial protective role in filtering and absorbing UV radiation, preventing deep penetration, and reducing oxidative stress in the skin. It functions as a biophysical UV filter, dissipating up to 99.9% of absorbed UV radiation as heat, thereby reducing direct DNA and cellular damage [5]. Furthermore, melanin has been shown to act as a biochemical antioxidant, neutralizing free radicals generated during UV exposure [6].

In vitiligo, the complete absence of functional melanocytes in depigmented areas leads to an exaggerated susceptibility to UV‐induced damage. Lacking this intrinsic defense, affected skin experiences increased penetration of UV photons, resulting in a heightened production of ROS. These ROS can damage cellular components, including DNA, proteins, and lipids, triggering apoptotic pathways and inflammatory cascades [7]. The resulting oxidative stress plays a pivotal role in both disease pathogenesis and exacerbation of cutaneous inflammation. Inflammatory mediators, including tumor necrosis factor‐alpha (TNF‐α), Interleukin 6 (IL‐6), and interferon‐gamma (IFN‐γ), further promote keratinocyte apoptosis and immune cell recruitment, exacerbating photodamage and increasing the likelihood of photosensitivity dermatitis [9].

## 5. Mechanism of Thiazide‐Induced Photosensitivity and Its Impact on Vitiligo

Thiazide diuretics, including HCTZ, are well‐documented photosensitizing agents that can trigger both phototoxic and photoallergic cutaneous reactions. Their photosensitizing properties arise from their ability to absorb UV radiation, generate free radicals, and induce oxidative cellular damage [10]. This is particularly relevant in individuals with vitiligo, where the absence of melanin allows greater UV penetration, amplifying the cytotoxic effects of drug‐induced ROS formation.

The pathophysiology of thiazide‐induced phototoxicity is thought to involve several key mechanisms:

1. UV Absorption and Excitation: Thiazide diuretics absorb UVA and UVB radiation, leading to electron excitation and the formation of high‐energy intermediates [4].
2. Generation of ROS and Lipid Peroxidation: These excited intermediates interact with oxygen, producing ROS such as superoxide anions (${\text{O}}_{2}^{-}$), hydrogen peroxide (H_2_O_2_), and hydroxyl radicals (OH·). These ROS, in turn, initiate lipid peroxidation, damaging keratinocyte membranes, mitochondrial DNA, and essential structural proteins [8].
3. Apoptosis of Keratinocytes and Localized Inflammation: The cumulative cellular damage triggers programmed cell death (apoptosis) in keratinocytes. Additionally, proinflammatory mediators such as IL‐1β, IL‐8, and Prostaglandin E2 (PGE2) further contribute to the erythema, pruritus, and papular eruptions characteristic of drug‐induced photosensitivity [2].

Given these mechanistic insights, individuals with vitiligo face a dual vulnerability: The absence of melanin allows for unimpeded UV penetration, while photosensitizing medications such as thiazide diuretics amplify UV‐induced damage, leading to exaggerated phototoxic responses in depigmented skin.

## 6. Clinical Implications and Management Strategies

Identifying pharmacologic photosensitizers in patients with vitiligo is critical to prevent unnecessary exacerbation of cutaneous damage. A comprehensive medication history should be obtained in all patients presenting with new‐onset photosensitive eruptions, with particular attention to antihypertensives (thiazides and calcium channel blockers), antibiotics (fluoroquinolones and tetracyclines), NSAIDs, and retinoids, which are well‐established photosensitizing agents.

The primary management approach involves the following.

1. Drug Discontinuation or Substitution: If feasible, switching to a nonphotosensitizing antihypertensive (e.g., angiotensin receptor blockers without HCTZ) should be considered [11].
2. Strict Photoprotection: Patients should use broad‐spectrum sunscreens (SPF 50+, with UVA/UVB coverage), UV‐protective clothing, and wide‐brimmed hats to minimize direct UV exposure [12].
3. Topical and Systemic Therapies: Topical corticosteroids or calcineurin inhibitors (e.g., tacrolimus) can be employed to reduce inflammatory responses in cases of persistent reactions. Severe or widespread cases may require short‐term systemic corticosteroids or antihistamines for symptomatic relief [2].
4. Patient Education: Counseling on UV avoidance, proper sunscreen application, and self‐monitoring for cutaneous changes is crucial to prevent recurrent drug‐induced photosensitivity reactions.

This case underscores the importance of early recognition and intervention in preventing worsening skin reactions in photosensitive populations such as patients with vitiligo. Given the growing evidence of oxidative stress in vitiligo pathogenesis, ongoing research into the interplay between phototoxic agents and depigmented skin may yield new insights into targeted therapeutic approaches.

## 7. Future Directions and Research Considerations

While the mechanistic relationship between photosensitizing drugs, UV radiation, and oxidative stress is well‐documented, gaps remain in understanding the long‐term implications of repeated phototoxic injury in patients with vitiligo.

Future studies should focus on elucidating molecular pathways linking photosensitizing drugs to keratinocyte apoptosis in depigmented skin, developing novel antioxidant therapies to mitigate UV‐induced oxidative damage in vitiligo patients at high risk for photosensitivity, and exploring genetic and epigenetic factors that may predispose certain individuals to heightened drug‐induced phototoxicity. By integrating dermatological pharmacology, photobiology, and immunopathology, clinicians can better anticipate and prevent adverse cutaneous reactions in photosensitive populations. This will ultimately improve the quality of life and long‐term skin health of individuals with vitiligo.

## Consent

No written consent has been obtained from the patient, as there are no patient‐identifiable data included in this case report.

## Conflicts of Interest

The authors declare no conflicts of interest.

## Funding

No funding was received for this manuscript.

## Data Availability

The data that support the findings of this study are available from the corresponding author upon reasonable request.
